# Economic evaluation of the second-line regimen of liposome irinotecan (II) combined with 5-FU/LV versus placebo combined with 5-FU/LV for locally advanced or metastatic pancreatic ductal adenocarcinoma in China

**DOI:** 10.1371/journal.pone.0351853

**Published:** 2026-06-22

**Authors:** Jinlong Huang, Hanyun Ye, Jingyang Lin, Dan Luo, Ping Huang, Xiaochun Zheng

**Affiliations:** 1 Center for Clinical Pharmacy, Cancer Center, Department of Pharmacy, Zhejiang Provincial People’s Hospital (Affiliated People’s Hospital), Hangzhou Medical College, Hangzhou, Zhejiang, China; 2 School of Pharmacy, Hangzhou Normal University, Hangzhou, Zhejiang, China; 3 Heart Center, Department of Cardiovascular Medicine, Zhejiang Provincial People’s Hospital (Affiliated People’s Hospital), Hangzhou Medical College, Hangzhou, Zhejiang, China; Goethe University Hospital Frankfurt, GERMANY

## Abstract

**Background:**

The cost-effectiveness of liposome irinotecan (II) (HR070803) in combination with 5-fluorouracil and leucovorin as a treatment for patients with locally advanced or metastatic pancreatic ductal adenocarcinoma offering a potential new standard of care has not been established. Considering the high cost of liposome irinotecan (II), the aim of this study was to evaluate the economic value of liposome irinotecan (II) combined with 5-fluorouracil and leucovorin（5-FU/LV） versus placebo combined with 5-FU/LV for this indication from the perspective of the Chinese healthcare system.

**Methods:**

We developed a three-state Markov model based on the trial: NCT05074589 to estimate lifetime costs, quality-adjusted life-years (QALYs), and incremental cost-effect ratios (ICERs) in terms of cost per QALY gained. The utility of health status and the disutility of adverse events were obtained from the published literature. Costs were obtained from local hospitals and published literature. Costs and outcomes were discounted at a discount rate of 5% per year. To assess the robustness of the model, univariate and probabilistic sensitivity analysis was performed.

**Results:**

In the base-case analysis, liposome irinotecan (II) regimen provided an additional 0.08 QALYs compared to the placebo regimen with an ICER of $310,418.81/ QALY gained, which indicates that the liposome irinotecan (II) regimen is not cost-effective at the $39,221.95/ QALY threshold. One-way sensitivity analyses showed that the model was most sensitive to the utility of PFS, the cost of liposome irinotecan (II), and the utility of PD. Probabilistic sensitivity analyses showed that the liposome irinotecan (II) regimen had a probability of 0 for having a cost effect at $39,221.95/ QALY. Price simulations show that the liposome irinotecan (II) option is cost-effective at a willingness-to-pay (WTP) of $39,221.95/QALY if the price of liposome irinotecan (II) is reduced to $2.93/mg (88.6% reduction).

**Conclusions:**

From the perspective of the Chinese healthcare system, liposome irinotecan (II) in combination with 5-FU/LV was less cost-effective than placebo in combination with 5-FU/LV for locally advanced or metastatic pancreatic ductal adenocarcinoma.

## Introduction

Pancreatic cancer is currently the seventh leading cause of cancer-related deaths globally and the fourth leading cause after lung, colorectal, and breast cancers. By 2030, it is projected to become the third leading cause [1–4]. Due to its insidious nature, most patients present with advanced or metastatic disease. For those diagnosed with metastatic disease, the 5-year survival rate is less than 10%, with an estimated 5-year survival rate of only 3% [5]. Pancreatic ductal adenocarcinoma (PDAC) is the most common pathological type of pancreatic cancer, accounting for approximately 90% of cases. This underscores the urgent need for more effective treatments. Currently, the standard of care for treatment-naive patients with locally advanced or metastatic PDAC includes chemotherapeutic regimens such as AG (nab-paclitaxel plus gemcitabine) and FOLFIRINOX (a combination of leucovorin (LV), 5-fluorouracil (5-FU), irinotecan hydrochloride, and oxaliplatin) [6,7]. Although these therapies are initially effective, most patients eventually experience disease progression or relapse, highlighting the need for second-line treatment strategies following first-line therapy [8].

Liposome irinotecan represents a significant advancement in pancreatic cancer treatment by utilizing nanotechnology to encapsulate the drug. This encapsulation prevents premature degradation, promotes tumor accumulation, and reduces systemic toxicity [9]. In recent years, liposome irinotecan (II), HR070803, was developed and approved for marketing in December 2023 in China based on an innovative modification of the technology platform of existing commercialized liposome drugs. Liposome irinotecan (II) is a structurally optimized irinotecan hydrochloride nano-delivery system, a formulation that employs next-generation nano-liposome engineering technology to significantly improve the physicochemical properties of the drug delivery system through the integration of surface-functionalized modified polyethylene glycol-phospholipid complexes [10]. Compared to marketed products like Onivyde, liposome irinotecan (II) features an optimized particle size of 80–90 nm. This is significantly smaller than the > 100 nm range typical of traditional liposomes (p < 0.05) [11]. Precisely controlled nanoscale parameters enhance passive tumor targeting, while a reduced maximum plasma concentration improves the therapeutic index and attenuates dose-related toxicities [12].

A subsequent randomized phase III study, PAN-HEROIC-1 (NCT05074589) [13] evaluated the efficacy and safety of liposome irinotecan (II) in combination with 5-FU/LV for the treatment of locally advanced or metastatic PDAC. The study found that liposome irinotecan (II) combined with 5-FU/LV significantly improved OS compared to placebo plus 5-FU/LV (median, 7.4 months [95% CI 6.1–8.4] versus 5.0 months [95% CI 4.3–6.0]; hazard ratio [HR], 0.63 [95% CI 0.48–0.84]; log-rank P = 0.002). Progression-free survival (PFS) also showed improvement with liposome irinotecan (II) plus 5-FU/LV compared to placebo plus 5-FU/LV (median: 4.2 months [95% CI 2.9–5.6] versus 1.5 months [95% CI 1.4–1.6]; HR, 0.36 [95% CI 0.27–0.48]; log-rank P < 0.001). Based on these results, liposome irinotecan (II) in combination with 5-FU/LV was approved by the National Medical Products Administration of China in January 2024 for the second-line treatment of locally advanced or metastatic PDAC.

The clinical efficacy of liposome irinotecan (II) represents a significant therapeutic advancement; however, clinical utility must be weighed against economic reality. With the rising incidence of pancreatic cancer in China, the aggregate cost of treating PDAC is escalating, placing a strain on the healthcare budget. To ensure the sustainability of the social health insurance system, it is crucial to determine whether the survival benefits offered by liposome irinotecan (II) translate into “value for money” compared to standard therapies. There is currently a paucity of evidence regarding the economic implications of liposome irinotecan (II) in the Chinese setting. Therefore, to bridge this gap and assist regulatory bodies in making informed coverage decisions, this study aimed to evaluate the economic value of liposome irinotecan (II) for the treatment of locally advanced or metastatic PDAC from the perspective of the Chinese healthcare system.

### Methods

#### Study design.

This study adhered to the Consolidated Health Economic Evaluation Reporting Standards reporting guideline (S1 File) [14]. Since the analysis was based exclusively on aggregated data from published studies with no direct involvement of human subjects, formal institutional ethics approval and informed consent were waived by the Institutional Review Board of Zhejiang Provincial People’s Hospital. The target population consisted of patients with unresectable, locally advanced or metastatic PDAC from China who had received prior gemcitabine-based therapy. These patients were assumed to have similar baseline characteristics to those enrolled in the PAN-HEROIC-1 study (NCT05074589) trial (see S1 Table for details of baseline information). In addition, our model assumed a patient cohort with a body surface area (BSA) of 1.72 m^2^. Patients were assigned to either the liposome irinotecan (II) regimen or the placebo regimen. Patients were randomized 1:1 to receive either liposome irinotecan (II) (56.5 mg/m^2^) or placebo, both in combination with 5-FU (2000 mg/m^2^) and LV (200 mg/m^2^). All given patients continue their respective regimens until progression, uncontrolled toxicity, or death is observed.

#### Model structure.

**Fig 1:**
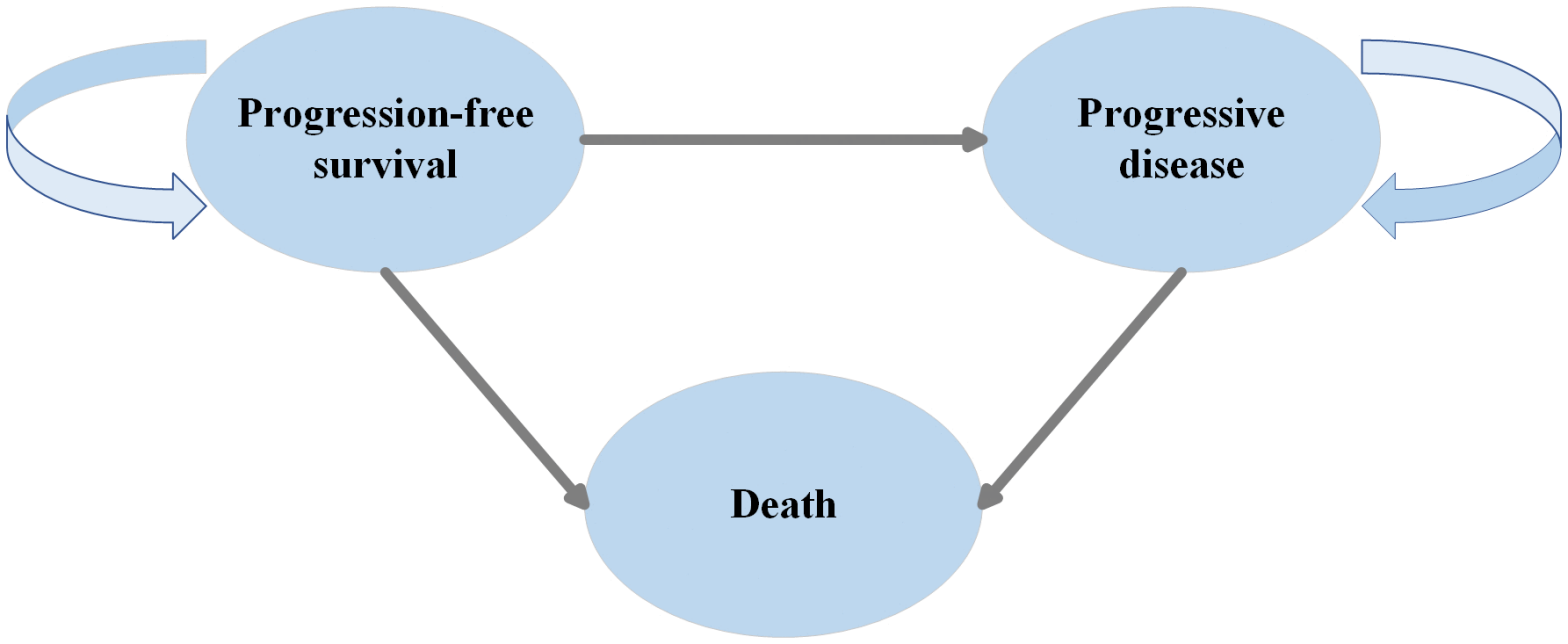
Markov model structure.

We developed a decision-analytic Markov model to compare the cost-effectiveness of the liposome irinotecan (II) regimen with a placebo regimen as a second-line treatment for locally advanced or metastatic PDAC. The model includes three mutually exclusive health states: progression-free survival (PFS), progressive disease (PD), and death (Fig 1). The model’s time horizon is based on a lifetime perspective, with 99% of patients eventually transitioning to the death state. The cycle length is defined as one treatment cycle, which is 14 days. Our analysis was conducted from the perspective of the Chinese healthcare system.

The cost-effectiveness evaluation utilizes incremental cost-effectiveness ratios (ICERs) based on total costs and quality-adjusted life years (QALYs). Both costs and utilities were discounted at an annual rate of 5%. We set the willingness-to-pay (WTP) threshold at 1 ~ 3 times China’s gross domestic product (GDP) per capita, as recommended by China’s pharmacoeconomic evaluation guidelines [15]. In our model, the WTP was set to $39,221.95 per QALY, which is three times the per capita GDP of China in 2024. If the ICER was less than the WTP threshold, the liposome irinotecan (II) regimen was considered superior compared with the placebo regimen. Otherwise, the liposome irinotecan (II) regimen was not cost-effective.

Our modeling was performed using Treeage Pro Suit 2022. Individual patient data (IPD) were reconstructed and survival outcomes extrapolated using the “flexsurv” and “survHE” packages of R software, and plotted using “ggplot2” of R software. All analyses were based on R software, Treeage Pro Suit 2022 and Microsoft Excel 2021.

#### Effectiveness.

Survival curve data were extracted using GetData Graph Digitizer (version 2.26). Individual patient data (IPD) were reconstructed using R software to plot Kaplan-Meier survival curves for progression-free survival (PFS) and overall survival (OS). Six distributions were applied for curve fitting and extrapolation: Exponential, Weibull, Gamma, Gompertz, Log-logistic, and Log-normal. The goodness of fit was assessed using the Akaike information criterion (AIC) and visual inspection. Lower AIC values, combined with visual assessment, indicated a better fit for the selected model. The best-fit distributions are presented in Table 1, and the AIC values for each distribution are detailed in S2 Table. The results of the survival curve simulation are shown in S1 Fig.

**Table 1:** Key parameters.

Parameters	Value	Range	Distribution	Reference
**Clinical data**				
**Log-normal model of PFS for liposomal irinotecan (II) regimen**	μ=0.998, σ=0.888			[13]
**Log-logistic model of PFS for Placebo regimen**	λ=2.970, γ=1.678			[13]
**Log-logistic model of OS for liposomal irinotecan (II) regimen**	λ=1.993, γ=6.156			[13]
**Log-normal model of OS for Placebo regimen**	μ=1.658, σ=0.822			[13]
**Cost ($)**				
**Liposomal irinotecan (II) (37.7 mg)**	968.87	775.10-1162.64	Gamma	Local hospital
**5-fluorouracil (250 mg)**	4.1	3.28-4.92	Gamma	Local hospital
**Leucovorin (100 mg)**	3.5	2.8-4.2	Gamma	Local hospital
**CT**	25.18	20.14-30.22	Gamma	Local hospital
**Laboratory test**	104.82	83.86-125.78	Gamma	Local hospital
**Outpatient fees**	6.27	5.02-7.52	Gamma	Local hospital
**Cost of PD**	1064.65	851.72-1277.58	Gamma	[16]
**Cost of Grade>3 AEs in liposomal irinotecan (II) group**	36.34	29.07-43.61	Gamma	Local hospital
**Cost of Grade>3 AEs in Placebo group**	13.56	10.85-16.27	Gamma	Local hospital
**AEs incidence of liposomal irinotecan (II) group (%)**				
**Nausea**	1.40	1.12-1.68	Beta	[13]
**Vomiting**	4.80	3.84-5.76	Beta	[13]
**Asthenia**	4.10	3.28-4.92	Beta	[13]
**Decreased appetite**	2.70	2.16-3.24	Beta	[13]
**Diarrhea**	4.10	3.28-4.92	Beta	[13]
**Anemia**	6.10	4.88-7.32	Beta	[13]
**Neutrophil count decreased**	12.90	10.32-15.48	Beta	[13]
**Weight decreased**	0.70	0.56-0.84	Beta	[13]
**White blood cell count decreased**	8.20	6.56-9.84	Beta	[13]
**Alanine aminotransferase increased**	4.10	3.28-4.92	Beta	[13]
**Aspartate aminotransferase increased**	1.40	1.12-1.68	Beta	[13]
**Gamma-glutamyltransferase increased**	19.10	15.28-22.92	Beta	[13]
**Abdominal pain**	4.10	3.28-4.92	Beta	[13]
**Hypokalaemia**	4.80	3.84-5.76	Beta	[13]
**Abdominal pain upper**	0.70	0.56-0.84	Beta	[13]
**Blood bilirubin increased**	7.50	6.00-9.00	Beta	[13]
**Blood alkaline phosphatase increased**	4.10	3.28-4.92	Beta	[13]
**Hyponatremia**	2.70	2.16-3.24	Beta	[13]
**Lymphocyte count decreased**	1.40	1.12-1.68	Beta	[13]
**Abdominal distension**	0.70	0.56-0.84	Beta	[13]
**AEs incidence of Placebo group (%)**				
**Vomiting**	2.00	1.60-2.40	Beta	[13]
**Asthenia**	2.00	1.60-2.40	Beta	[13]
**Decreased appetite**	1.30	1.04-1.56	Beta	[13]
**Diarrhea**	2.70	2.16-3.24	Beta	[13]
**Anemia**	2.70	2.16-3.24	Beta	[13]
**Weight decreased**	0.70	0.56-0.84	Beta	[13]
**White blood cell count decreased**	0.70	0.56-0.84	Beta	[13]
**Alanine aminotransferase increased**	2.00	1.60-2.40	Beta	[13]
**Aspartate aminotransferase increased**	1.30	1.04-1.56	Beta	[13]
**Gamma-glutamyltransferase increased**	11.40	9.12-13.68	Beta	[13]
**Constipation**	0.70	0.56-0.84	Beta	[13]
**Hypoalbuminemia**	1.30	1.04-1.56	Beta	[13]
**Abdominal pain**	3.40	2.72-4.08	Beta	[13]
**Pyrexia**	0.70	0.56-0.84	Beta	[13]
**Hypokalaemia**	2.70	2.16-3.24	Beta	[13]
**Back pain**	2.70	2.16-3.24	Beta	[13]
**Abdominal pain upper**	0.70	0.56-0.84	Beta	[13]
**Blood bilirubin increased**	5.40	4.32-6.48	Beta	[13]
**Blood alkaline phosphatase increased**	3.40	2.72-4.08	Beta	[13]
**Hyponatremia**	2.70	2.16-3.24	Beta	[13]
**Lymphocyte count decreased**	1.30	1.04-1.56	Beta	[13]
**Utilities**				
**PFS**	0.74	0.60-0.89	Beta	[17,18]
**PD**	0.67	0.54-0.80	Beta	[17,18]
**Disutility due to AEs (grade >3) in liposomal irinotecan (II) group**	0.13	0.10-0.16	Beta	[16–23]
**Disutility due to AEs (grade >3) in Placebo group**	0.08	0.06-0.10	Beta	[16–23]
**BSA**	1.72	1.38-2.06	Normal	Expert Opinion
**Discount (%)**	5.00	0.00-8.00	Beta	[15]

OS = Overall survival; PFS = Progression-free survival; PD = Progressive disease; AEs = Adverse events.

#### Cost and utility.

In our economic model, we considered only direct healthcare costs from the perspective of the Chinese healthcare system. These costs included drug expenses, management costs for adverse reactions (grade ≥3), laboratory tests, radiology tests, outpatient visits, and treatment costs following disease progression. Carbohydrate antigen 19−9 (CA19−9) levels were measured every 6 weeks, assuming that patients were assessed for tumor response by CT or MRI every 6 weeks, in accordance with the NCT05074589 trial protocol and clinical practice. Treatment-related grade 3/4 adverse events included increased gamma-glutamyltransferase, decreased neutrophil count, increased blood bilirubin, and increased conjugated bilirubin. The administration costs and prices of drugs used for adverse events (AEs) were obtained from the Zhejiang Provincial People’s Hospital. We assumed that all AEs occurred in the first treatment cycle. And patients received best supportive care (BSC) in PD status. BSC data was obtained from previously published literature [16]. All the costs were converted into US dollar calculation with an annual exchange rate of 2024 (1.00 US dollar = 7.12 Yuan).

The utility values for the different health states were obtained from previously published literature, where the utility values were taken as 0.74 for the PFS period and 0.67 for the PD period [17,18]. It is assumed that the utility values for PFS and PS status are the same regardless of the treatment. In our study, we considered disutility values associated with adverse effects, derived from previous studies (S3 Table) [16–23]. The model input parameters are shown in Table 1.

#### Sensitivity analysis.

To assess the robustness of the analytical results, one-way deterministic sensitivity analysis (DSA) and probabilistic sensitivity analysis (PSA) were conducted to determine the impact of each independent variable on the results. For DSA, inputs included a reasonable change (±20%) within the estimated range of the underlying price or a 95% confidence interval (CI) for the parameter. The results of the DSA are presented in a tornado diagram.

PSA was performed using Monte Carlo simulations with 5,000 random individuals. We selected the gamma distribution for cost parameters and the beta distribution for utility values, proportions, and probability parameters. The cost-effectiveness acceptability curve (CEAC) was employed to assess the cost-effectiveness of the two treatment regimens across various WTP threshold ranges. Further analysis was conducted at WTP thresholds of 1× and 2 × the GDP per capita, in accordance with the China Pharmacoeconomic Evaluation Guidelines. The PSA results are presented in acceptability curves and scatter plots.

#### Price simulation.

Given the high cost of liposome irinotecan (II), we conducted price simulations, varying the price of liposome irinotecan (II) (1 mg) between $1 and $30, to assess the likelihood of the liposome irinotecan (II) regimen being cost-effective at the Chinese WTP threshold of $39,221.95 per QALY.

#### Scenario analysis.

To ensure the robustness of the model conclusions, we conducted a scenario analysis with adjusted discount rates, thereby investigating the impact of different discount rates (0%, 3% and 8%) on the pharmacoeconomic outcomes.

### Results

#### Base case analysis.

The results of the base case analysis are shown in Table 2. The liposome irinotecan (II) regimen had a lifetime cost of treatment of $38,157.19, which was higher than the placebo regimen’s lifetime cost of $11,969.54. The liposome irinotecan (II) regimen resulted in an increase of 0.08 QALYs compared with the placebo regimen. However, compared to the placebo regimen, the ICER for liposome irinotecan (II) was $310, 418.80/ QALY gained, which is well above the WTP threshold of $39,221.95/ QALY, demonstrating that the liposome irinotecan (II) regimen is not cost-effective compared to the placebo regimen.

**Table 2:** Results of base case analysis and scenario analysis.

Scenario	Treatment	Total cost ($)	QALY	Incremental	Incremental	ICER
				cost ($)	QALY	($/QALY)
**Base-case analysis**	HR070803 regimen	38,157.19	0.51			
	Placebo regimen	11,969.54	0.42	26,187.65	0.09	310,418.81
**0% discount**	HR070803 regimen	39,184.79	0.51			
	Placebo regimen	12,381.23	0.43	26,803.56	0.08	342,767.64
**3% discount**	HR070803 regimen	38,558.13	0.50			
	Placebo regimen	12,130.73	0.47	26,427.40	0.07	359,260.94
**8% discount**	HR070803 regimen	37,579.31	0.48			
	Placebo regimen	11,735.97	0.42	25,843.34	0.06	387,910.27

ICER: Incremental cost-effectiveness ratio

QALY: Quality-adjusted life year

#### Sensitivity analysis.

**Fig 2:**
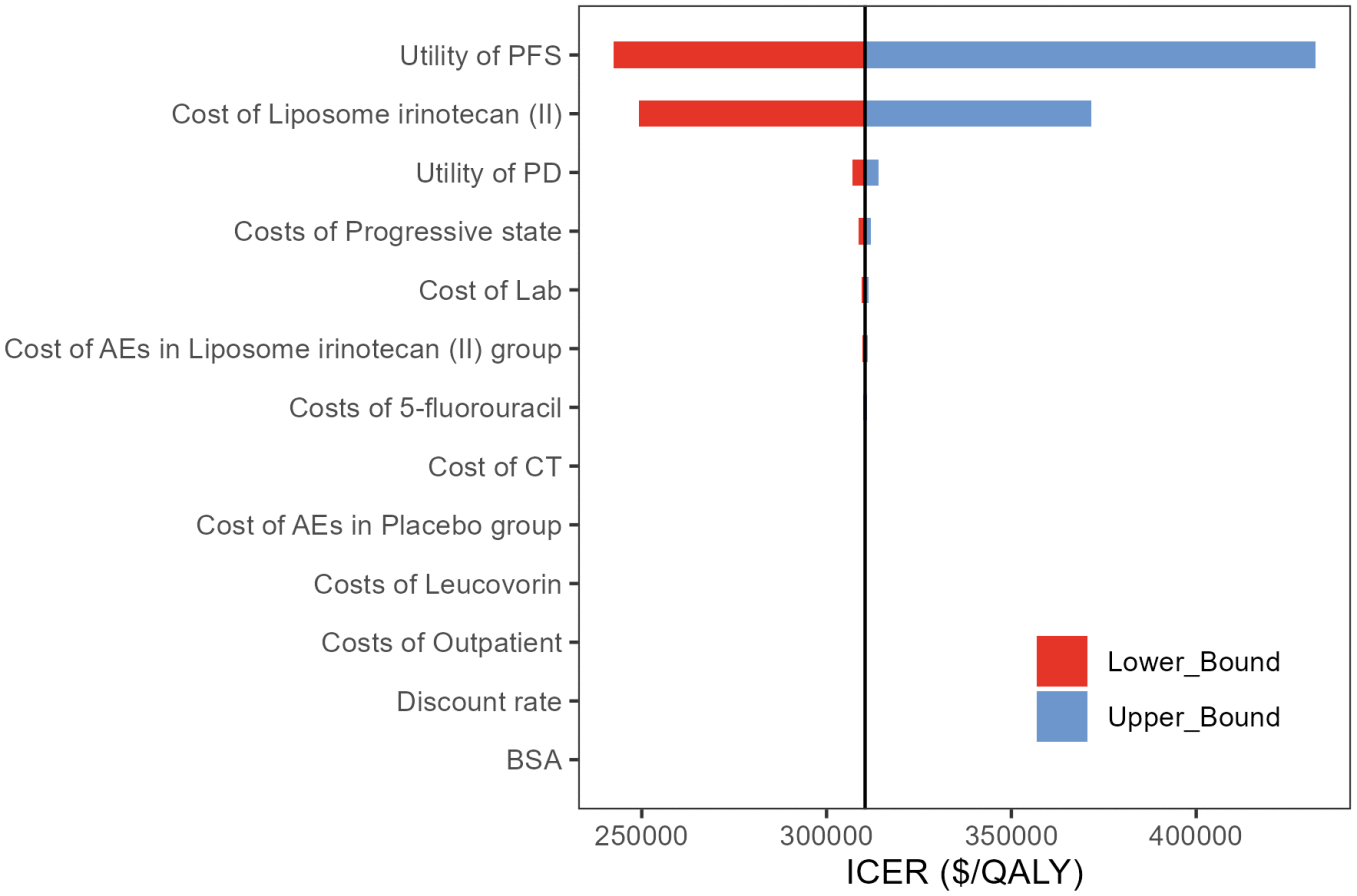
Tornado diagrams of univariable sensitivity analysis. PFS: Progression-free Survival; PD: Progress Disease; ICERs: Incremental Cost-effect Ratios; QALYs: Quality-adjusted Life-years; BSA: Body Surface Area.

**Fig 3:**
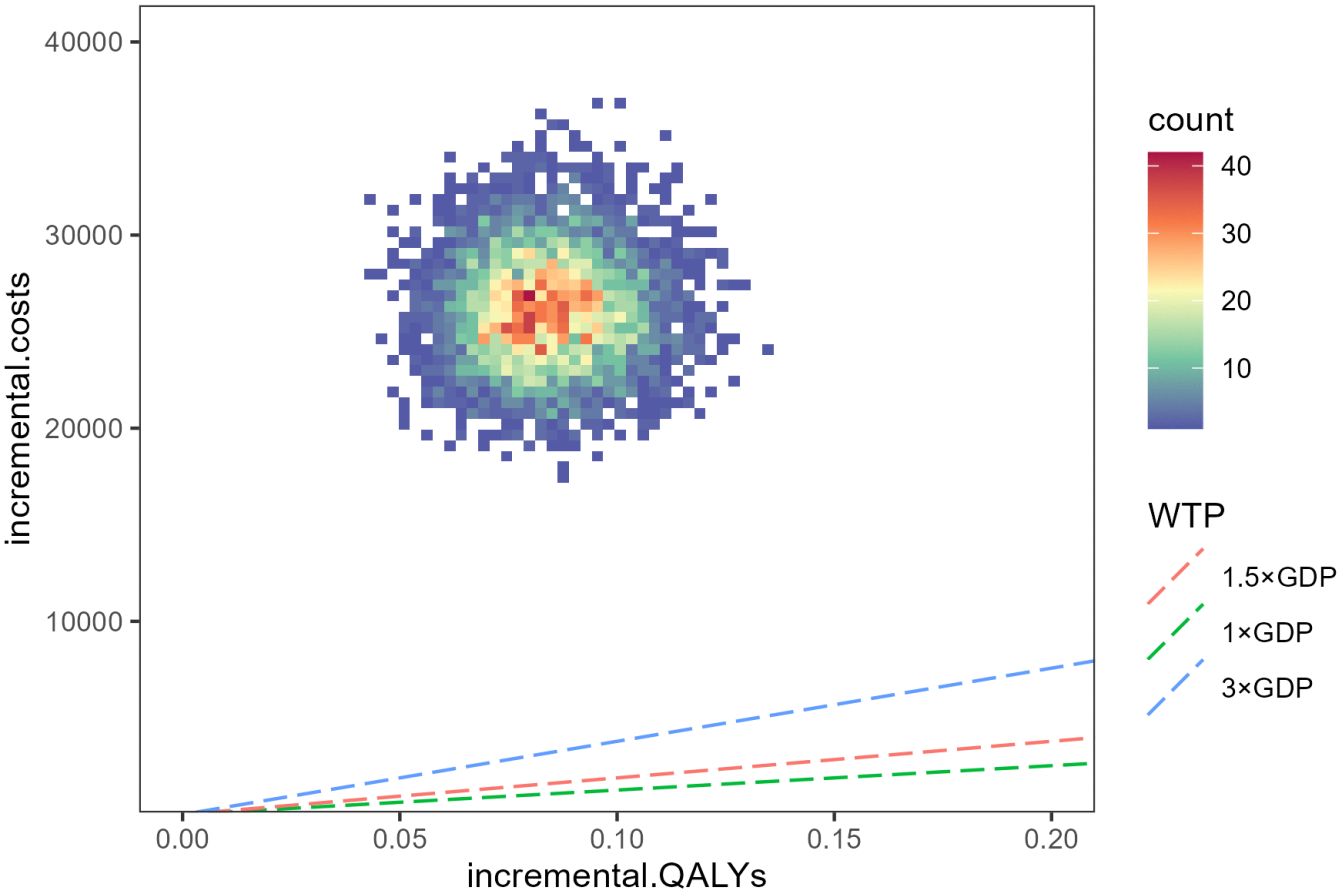
Scatter plots of 5000 Monte Carlo simulated patients. QALYs: Quality-adjusted Life-years; WTP: Willingness-to-pay.

**Fig 4:**
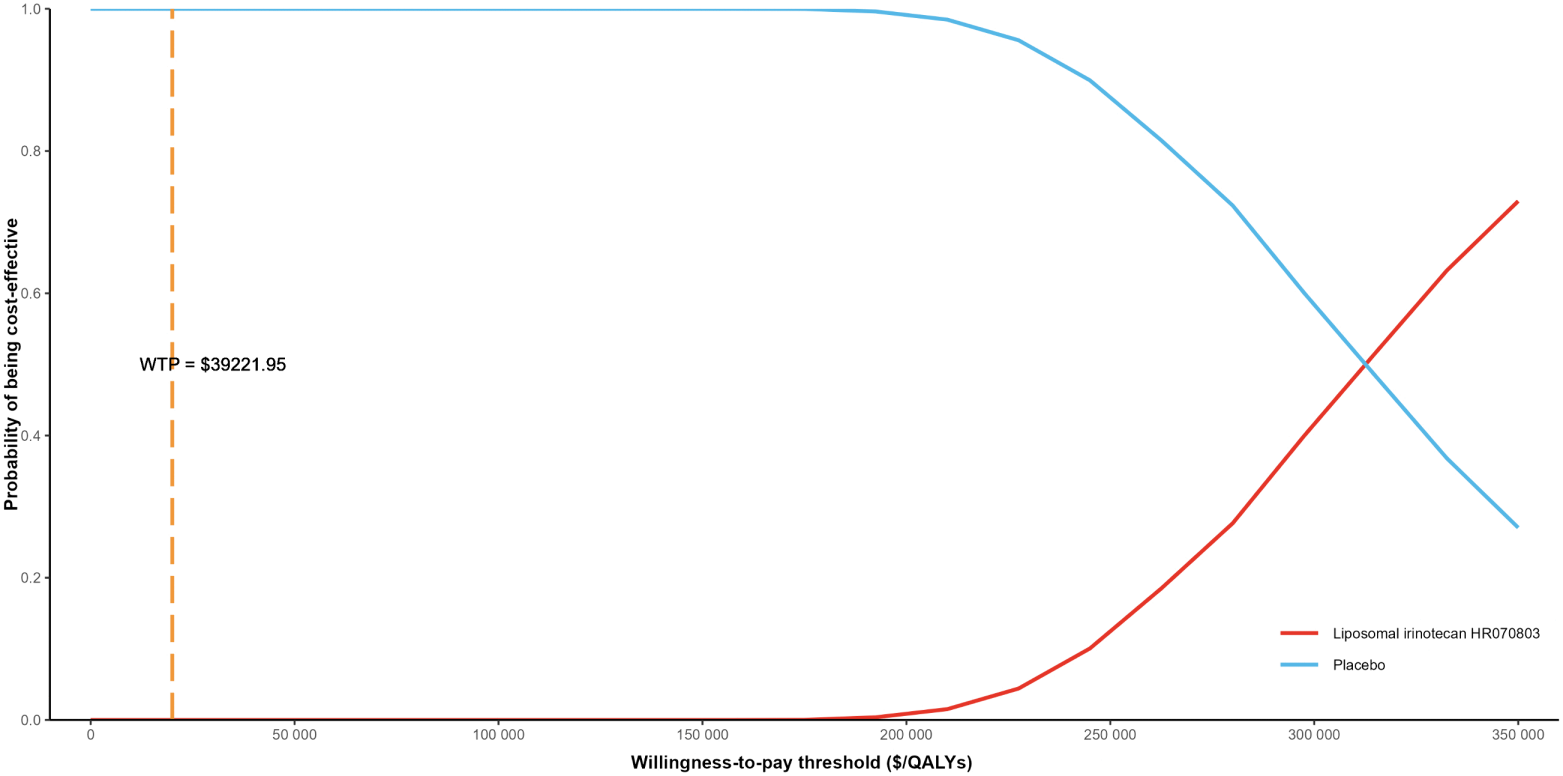
The cost-effectiveness acceptability curve. QALYs: Quality-adjusted Life-years; WTP: Willingness-to-pay.

The DSA results are shown in Fig 2. The main factors that were identified as influencing ICER were the utility of PFS, the cost of liposome irinotecan (II) and the utility of PD. However, despite changing these parameters, the ICER remains in the range of 242,296.39–432,377.50/ QALY for the utility of the PFS, and for the cost of liposome irinotecan (II), the ICER remains in the range of 249,167.86–371,669.75 / QALY range. In addition, the utility of PD, the cost of management of PD status, the cost of laboratory tests, and the cost of management of grade 3/4 adverse events occurring in the liposome irinotecan (II) group significantly affect the model. However, none of the parameter variations of these variables considered reducing the ICER below a predefined threshold of cost-effectiveness, and very few were cost-effective. Figures 3 and 4 show the probabilistic scatterplot and the acceptability curve for cost-effectiveness, respectively. The scatterplot of the probabilistic sensitivity analysis (Fig 3) shows that the ICERs of the 5000 Monte Carlo simulations were above the WTP threshold, which suggests that the liposome irinotecan (II) regimen is not cost-effective compared to the placebo regimen. The cost acceptability curve plot (Fig 4) shows that there is a 100.00% probability that group HR070803 will not be cost-effective when China sets a WTP value of $39,221.95.

#### Price simulation.

As the price of liposomal irinotecan decreased, the ICER values for the liposome irinotecan (II) regimen in combination with 5-fluorouracil and leucovorin gradually decreased relative to the placebo regimen. In the base case analysis, when the price of liposome irinotecan (II) was reduced by more than 88.6% (to less than $2.93/mg), the liposome irinotecan (II) regimen in combination with 5-fluorouracil and leucovorin became more cost-effective than the placebo in combination with 5-FU/LV regimen. Further, the liposome irinotecan (II) in combination with 5-fluorouracil and leucovorin regimen has a 99% probability of being cost-effective when the price reduction is greater than 94.2% (less than $ 1.49 / mg). The price simulation outcomes are displayed in S2 Fig.

#### Scenario analysis.

The results of the scenario analysis are summarized in Table 2. With the discount rate adjusted to 0%, 3% and 8%, the ICERs for the liposomal irinotecan (II) regimen were $342,767.64, $359,260.94 and $387,910.27, respectively. These findings consistently demonstrate that liposomal irinotecan (II) is not a cost-effective therapeutic option under either discount rate. Notably, the outcomes were relatively insensitive to variations in the discount rate, suggesting that only a substantial reduction in drug pricing would render the liposomal irinotecan (II) strategy economically viable.

## Discussion

The PAN-HEROIC-1 clinical trial demonstrated that liposome irinotecan (II) significantly improved PFS and OS in patients with locally advanced or metastatic PDAC. However, given the high cost of liposome irinotecan (II), an economic evaluation of its combination with the 5-FU/LV regimen is necessary. This is the first time that the cost-effectiveness of liposome irinotecan (II) as a second-line treatment option for locally advanced or metastatic PDAC by the Chinese healthcare system. In this study, we performed a Markov model-based cost-effectiveness analysis of the liposome irinotecan (II) combined with 5-FU/LV regimen versus placebo combined with 5-FU/LV regimen as a second-line treatment for locally advanced or metastatic PDAC in China, using available efficacy data from the PAN-HEROIC-1 trial and local cost data. Based on the results of our base case analysis, patients treated with liposome irinotecan (II) would receive an additional 0.08 QALYs and spend an additional $26,187.65, resulting in ICERs of $310,418.81 per QALY, which is higher than the threshold for WTP. The ICERs indicate that, assuming a WTP threshold of three times the GDP per QALY per capita of 39,221.95 $/QALY, liposome irinotecan (II) is not cost-effective.

In a study by Xiang Z et al [24], the cost-effectiveness of three regimens for the treatment of mPDAC, namely NALIRIFOX (liposomal irinotecan, oxaliplatin, leucovorin, and fluorouracil), FOLFIRINOX (leucovorin, fluorouracil, irinotecan and oxaliplatin), and GEMNABP (gemcitabine and nab-paclitaxel) regimens for the treatment of mPDAC are cost-effective, and the results of the study show that the GEMNABP regimen is the preferred choice for these three first-line combination chemotherapy regimens under current market conditions. The NALIRIFOX regimen was not cost-effective. In a previous study by Shao et al [18], which constructed a partitioned survival model based on the NAPOLI-3 trial to assess the cost-effectiveness of the NALIRIFOX (liposomal irinotecan in combination with fluorouracil, folinic acid protein, and oxaliplatin) regimen for the treatment of mPDAC from the perspective of the U.S. public payer. The results of the study by Shao et al showed that NALIRIFOX was not cost-effective even when a WTP of $150,000/QALY threshold was set. Our findings are consistent with previous studies that the liposome irinotecan (II) regimen, despite its clear clinical benefit, may be too expensive to be acceptable to patients.

Each country defines the WTP threshold differently, and different countries use different WTP values for healthcare cost-effectiveness analysis. The recommended QALY for the United States is $150,000–200,000 [25]. In the European region, the common cost-effectiveness thresholds used range from $100,000 to $150,000 per QALY [26]. The WTP thresholds commonly used in the Canadian health care system are $50,000 and $100,000 per QALY [27]. In Japan, the recommended WTP threshold is 7.5 million yen per QALY of ICER based on the Japanese cost-effectiveness evaluation system [28]. In China, the WTP threshold is set at three times the per capita GDP, following the standardized methodology outlined in the China Pharmacoeconomic Evaluation Guidelines. Despite recent skepticism regarding the use of three times GDP per capita as a cost-effectiveness threshold, it remains the most established, widely used, and standardized method for setting cost-effectiveness thresholds in China. Differences in health insurance types, coverage, and reimbursement rates exist across China’s provinces. Selecting cost-effective treatment options can optimize the allocation of health insurance resources. Our analysis reveals that liposome irinotecan (II) is not cost-effective under the 1x GDP and 1.5x GDP scenarios. Therefore, we recommend that health insurance authorities increase reimbursement rates for locally advanced or metastatic PDAC to enable more patients to benefit.

Our economic model has several limitations. First, the clinical data were sourced from the NCT05074589 trial, which may not fully reflect real-world outcomes. While we acknowledge that simplifying assumptions about post-treatment effects could oversimplify the results, modifying survival curves to address these factors was impractical within the scope of our analysis. Second, utility values in the model were derived from published health technology assessment literature, not the NCT05074589 trial. Assuming identical utility values for both patient groups may introduce bias, and varying utility inputs may also exert an impact on the results. Additionally, using utility values from external randomized controlled trials (RCTs) rather than the trial itself poses further limitations. Third, our study was based on real-world cost data from local hospitals. Although we conducted a sensitivity analysis of price parameters, variations in cost parameters could still affect the outcomes of the model. And our analysis exclusively considered costs and negative utility associated with grade 3 or higher AEs, potentially affecting modeling accuracy. Despite these limitations, this study offers a valuable reference for treatment decision-making by physicians and patients.

### Conclusion

From the perspective of the Chinese health service system, the liposome irinotecan (II) regimen is not a more cost-effective treatment option than placebo regimen for locally advanced or metastatic pancreatic ductal adenocarcinoma. However, price simulations showed that if the price of liposome irinotecan (II) is reduced to $2.93/mg, which represents an 88.6% decrease. The liposome irinotecan (II) in combination with the 5-FU/LV regimen at the WTP threshold of $39,221.95/QALY could achieve cost-effectiveness.

## Supporting information

S1 Fig Kaplan Meier curves and parametric model fitting. KM, Kaplan Meier; OS, Overall survival; PFS, Progression-free survival.
(TIF)

S2 Fig CEAC diagram for price simulation. CEAC, Cost-effectiveness acceptability curve.
(TIF)

S1 Table Baseline characteristics of patients in PAN-HEROIC-1 study. (DOCX)

S2 Table The Akaike information criteria (AIC). OS, Overall survival; PFS, Progression-free survival; AIC, Akaike information criterion; AFT, Accelerate Failure Time; PH, Proportional Hazards.
(DOCX)

S3 Table Specific disutility values for each adverse reaction. (DOCX)

S1 File Consolidated health economic evaluation reporting standards reporting guideline. (PDF)
