# Supplementary figures and images for: Economic evaluation of the second-line regimen of liposome irinotecan (II) combined with 5-FU/LV versus placebo combined with 5-FU/LV for locally advanced or metastatic pancreatic ductal adenocarcinoma in China
Source: PLoS One. 2026 Jun 22;21(6):e0351853. doi: 10.1371/journal.pone.0351853 (PMC13286221; doi:10.1371/journal.pone.0351853)

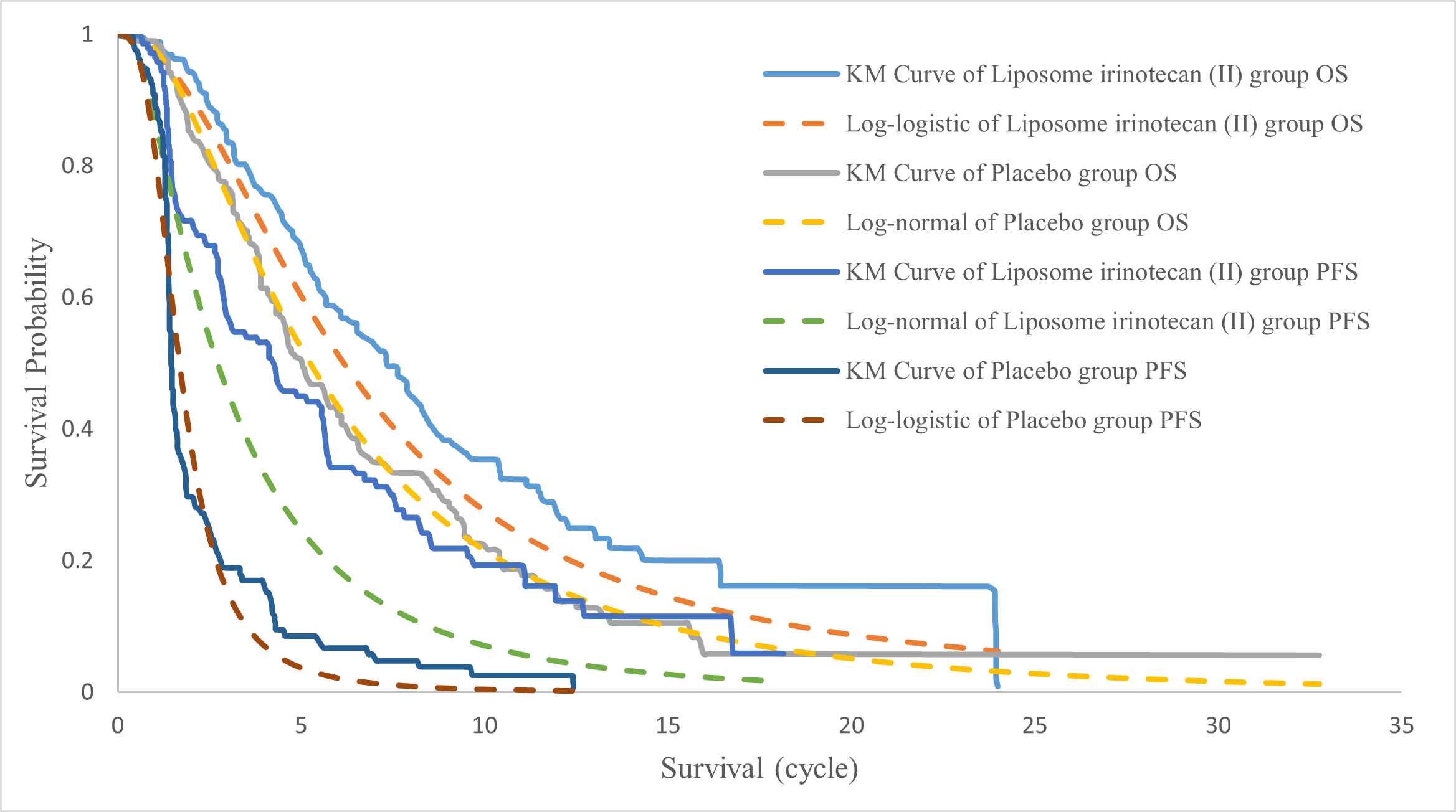

Supplement: S1 Fig — KM, Kaplan Meier; OS, Overall survival; PFS, Progression-free survival. (TIF) [file pone.0351853.s001.tif]
