# Supplementary material for: Economic evaluation of the second-line regimen of liposome irinotecan (II) combined with 5-FU/LV versus placebo combined with 5-FU/LV for locally advanced or metastatic pancreatic ductal adenocarcinoma in China
Source: PLoS One. 2026 Jun 22;21(6):e0351853. doi: 10.1371/journal.pone.0351853 (PMC13286221; doi:10.1371/journal.pone.0351853)
Supplement: S1 Table — (DOCX) [file pone.0351853.s003.docx]

**S1 Table. Baseline characteristics of patients in PAN-HEROIC-1 study**

|  | **liposome irinotecan (II) group (n = 149)** | **Placebo group (n = 149)** |
| --- | --- | --- |
| **Age, years** | 60 (52–66) | 59 (54–65) |
| **Male** | 95 (63.8) | 93 (62.4) |
| **ECOG performance status** |  |  |
| **0** | 25 (16.8) | 26 (17.5) |
| **1** | 124 (83.2) | 123 (82.6) |
| **Pancreatic tumor location** |  |  |
| **Head** | 60 (40.3) | 65 (43.6) |
| **Body** | 6 (4.0) | 7 (4.7) |
| **Tail** | 62 (41.6) | 59 (39.6) |
| **Multicentric** | 21 (14.1) | 18 (12.1) |
| **Clinical TNM stage** |  |  |
| **Stage II-III (locally advanced)** | 5 (3.4) | 6 (4.0) |
| **Stage IV (metastatic)** | 144 (96.6) | 142 (95.3) |
| **Site of metastatic lesions** |  |  |
| **Liver** | 110 (73.8) | 100 (67.1) |
| **Lung** | 38 (25.5) | 40 (26.9) |
| **Lymph node, regional** | 49 (32.9) | 44 (29.5) |
| **Lymph node, distant** | 42 (28.2) | 55 (36.9) |
| **Peritoneum** | 21 (14.1) | 12 (8.1) |
| **Other** | 54 (36.2) | 55 (36.9) |
| **Number of metastatic sites** |  |  |
| **0** | 1 (0.7) | 3 (2.0) |
| **1** | 40 (26.8) | 45 (30.2) |
| **2** | 58 (38.9) | 49 (32.9) |
| **3** | 33 (22.1) | 33 (22.1) |
| **≥ 4** | 17 (11.4) | 19 (12.8) |
| **CA19-9, U/mL** | 267.3 (64.5–1523.5) | 503.8 (68.6–2056.0) |
| **Albumin** |  |  |
| **< 40 g/L** | 45 (30.2) | 46(30.9) |
| **≥ 40 g/L** | 104 (69.8) | 103(69.1) |

This table was extracted from PAN-HEROIC-1 study.
