# Supplementary material for: Economic evaluation of the second-line regimen of liposome irinotecan (II) combined with 5-FU/LV versus placebo combined with 5-FU/LV for locally advanced or metastatic pancreatic ductal adenocarcinoma in China
Source: PLoS One. 2026 Jun 22;21(6):e0351853. doi: 10.1371/journal.pone.0351853 (PMC13286221; doi:10.1371/journal.pone.0351853)
Supplement: S2 Table — OS, Overall survival; PFS, Progression-free survival; AIC, Akaike information criterion; AFT, Accelerate Failure Time; PH, Proportional Hazards. (DOCX) [file pone.0351853.s004.docx]

**S2 Table. The Akaike information criteria (AIC)**

|  | **AIC value** | | | | | | |
| --- | --- | --- | --- | --- | --- | --- | --- |
| **Distribution** | Exponential | Gamma | Gompertz | Weibull (PH) | Loglogistic | Lognormal | Weibull (AFT) |
| **PFS** |  |  |  |  |  |  |  |
| **Liposome irinotecan (II) group** | 1108.86 | 1093.27 | 1108.32 | 1104.62 | 1039.81 | 1032.20 | 1104.62 |
| **Placebo group** | 477.24 | 439.40 | 479.11 | 459.41 | 390.68 | 401.99 | 459.41 |
| **OS** |  | | | | | | |
| **Liposome irinotecan (II) group** | 1455.54 | 1415.39 | 1450.49 | 1425.21 | 1403.40 | 1405.04 | 1425.21 |
| **Placebo group** | 750.38 | 728.48 | 749.95 | 735.40 | 720.02 | 717.54 | 735.40 |

Abbreviations:

OS, overall survival;

PFS, progression-free survival;

AIC, Akaike information criterion;

AFT, Accelerate Failure Time;

PH, Proportional Hazards.
