# Supplementary material for: Economic evaluation of the second-line regimen of liposome irinotecan (II) combined with 5-FU/LV versus placebo combined with 5-FU/LV for locally advanced or metastatic pancreatic ductal adenocarcinoma in China
Source: PLoS One. 2026 Jun 22;21(6):e0351853. doi: 10.1371/journal.pone.0351853 (PMC13286221; doi:10.1371/journal.pone.0351853)
Supplement: S3 Table — (DOCX) [file pone.0351853.s005.docx]

**S3 Table. Specific disutility values for each adverse reaction.**

| **Variables** | **disutility** | **Reference** |
| --- | --- | --- |
| **Nausea** | 0.05 | [16-23] |
| **Vomiting** | 0.05 | [16-23] |
| **Asthenia** | 0.204 | [16-23] |
| **Decreased appetite** | 0.002 | [16-23] |
| **Diarrhea** | 0.21 | [16-23] |
| **Anemia** | 0.204 | [16-23] |
| **Neutrophil count decreased** | 0.09 | [16-23] |
| **Weight decreased** | 0.17 | [16-23] |
| **White blood cell count decreased** | 0.17 | [16-23] |
| **Alanine aminotransferase increased** | 0.13 | [16-23] |
| **Aspartate aminotransferase increased** | 0.13 | [16-23] |
| **Gamma-glutamyltransferase increased** | 0.17 | [16-23] |
| **Constipation** | 0.17 | [16-23] |
| **Hypoalbuminemia** | 0.17 | [16-23] |
| **Abdominal pain** | 0.051 | [16-23] |
| **Pyrexia** | 0.17 | [16-23] |
| **Hypokalaemia** | 0.17 | [16-23] |
| **Back pain** | 0.13 | [16-23] |
| **Abdominal pain upper** | 0.051 | [16-23] |
| **Blood bilirubin increased** | 0.17 | [16-23] |
| **Blood alkaline phosphatase increased** | 0.17 | [16-23] |
| **Hyponatremia** | 0.17 | [16-23] |
| **Lymphocyte count decreased** | 0.17 | [16-23] |
| **Abdominal distension** | 0.13 | [16-23] |
