# Supplementary material for: Economic evaluation of the second-line regimen of liposome irinotecan (II) combined with 5-FU/LV versus placebo combined with 5-FU/LV for locally advanced or metastatic pancreatic ductal adenocarcinoma in China
Source: PLoS One. 2026 Jun 22;21(6):e0351853. doi: 10.1371/journal.pone.0351853 (PMC13286221; doi:10.1371/journal.pone.0351853)
Supplement: S1 File — (PDF) [file pone.0351853.s006.pdf]

CHEERS 2022 Checklist

Topic	No.	Item	Location where item is reported
**Title**			
	1	Identify the study as an economic evaluation and specify the interventions being compared.	Page1 Line 1
**Abstract**			
	2	Provide a structured summary that highlights context, key methods, results, and alternative analyses.	Page2 Line 27
**Introduction**			
**Background and objectives**	3	Give the context for the study, the study question, and its practical relevance for decision making in policy or practice.	Page4 Line 59
**Methods**			
**Health economic analysis plan**	4	Indicate whether a health economic analysis plan was developed and where available.	Page6 Line 116
**Study population**	5	Describe characteristics of the study population (such as age range, demographics, socioeconomic, or clinical characteristics).	Page6 Line 109
**Setting and location**	6	Provide relevant contextual information that may influence findings.	Page7 Line 125
**Comparators**	7	Describe the interventions or strategies being compared and why chosen.	Page2 Line 28,<br>Page2 Line 36
**Perspective**	8	State the perspective(s) adopted by the study and why chosen.	Page6 Line 117
**Time horizon**	9	State the time horizon for the study and why appropriate.	Page6 Line 121
**Discount rate**	10	Report the discount rate(s) and reason chosen.	Page7 Line 127
**Selection of outcomes**	11	Describe what outcomes were used as the measure(s) of benefit(s) and harm(s).	Page2 Line 36;<br>Page7 Line 125
**Measurement of outcomes**	12	Describe how outcomes used to capture benefit(s) and harm(s) were measured.	Page9 Line 174

Topic	No.	Item	Location where item is reported
**Valuation of outcomes**	13	Describe the population and methods used to measure and value outcomes.	Page9 Line 174
**Measurement and valuation of resources and costs**	14	Describe how costs were valued.	Page7 Line 149
**Currency, price date, and conversion**	15	Report the dates of the estimated resource quantities and unit costs, plus the currency and year of conversion.	Page8 Line 162
**Rationale and description of model**	16	If modelling is used, describe in detail and why used. Report if the model is publicly available and where it can be accessed.	Page6 Line 117;<br>Page17 Line 336
**Analytics and assumptions**	17	Describe any methods for analysing or statistically transforming data, any extrapolation methods, and approaches for validating any model used.	Page7 Line 138
**Characterising heterogeneity**	18	Describe any methods used for estimating how the results of the study vary for subgroups.	Page9 Line 189
**Characterising distributional effects**	19	Describe how impacts are distributed across different individuals or adjustments made to reflect priority populations.	Not applicable
**Characterising uncertainty**	20	Describe methods to characterise any sources of uncertainty in the analysis.	Page16 Line 203
**Approach to engagement with patients and others affected by the study**	21	Describe any approaches to engage patients or service recipients, the general public, communities, or stakeholders (such as clinicians or payers) in the design of the study.	Page10 Line 110
**Results**			
**Study parameters**	22	Report all analytic inputs (such as values, ranges, references) including uncertainty or distributional assumptions.	Page10 Line 196
**Summary of main results**	23	Report the mean values for the main categories of costs and outcomes of interest and summarise them in the most appropriate overall measure.	Page10 Line 195
**Effect of uncertainty**	24	Describe how uncertainty about analytic judgments, inputs, or projections affect findings. Report the effect of choice of discount rate and time horizon, if applicable.	Page10 Line 203

Topic	No.	Item	Location where item is reported
**Effect of engagement with patients and others affected by the study**	25	Report on any difference patient/service recipient, general public, community, or stakeholder involvement made to the approach or findings of the study	Not applicable
**Discussion**			
**Study findings, limitations, generalisability, and current knowledge**	26	Report key findings, limitations, ethical or equity considerations not captured, and how these could affect patients, policy, or practice.	Page11 Line 233
**Other relevant information**			
**Source of funding**	27	Describe how the study was funded and any role of the funder in the identification, design, conduct, and reporting of the analysis	Page16 Line 303
**Conflicts of interest**	28	Report authors conflicts of interest according to journal or International Committee of Medical Journal Editors requirements.	Page16 Line 334

From: Husereau D, Drummond M, Augustovski F, et al. Consolidated Health Economic Evaluation Reporting Standards 2022 (CHEERS 2022) Explanation and Elaboration: A Report of the ISPOR CHEERS II Good Practices Task Force. Value Health 2022;25. doi:10.1016/j.jval.2021.10.008
